# Periodontal treatment needs and systemic diseases in an older population in Greece

**DOI:** 10.4317/jced.52763

**Published:** 2016-02-01

**Authors:** Georgios S. Chatzopoulos, Lazaros Tsalikis

**Affiliations:** 1 DDS, Resident Advanced Education Program in Periodontology, School of Dentistry, University of Minnesota, USA; 2 DDS, Dr med dent, Associate Professor, Department of Preventive Dentistry, Periodontology and Implant biology, School of Dentistry, Aristotle University of Thessaloniki, Greece

## Abstract

**Background:**

To evaluate the relationship between systemic diseases, body mass index and periodontal treatment needs in an older population in Greece.

**Material and Methods:**

A total of 262 older people were clinically examined about their periodontal status and medical histories were recorded using a health history questionnaire. Additionally, weight and height measurements as well as demographic data were obtained from the participants in the study.

**Results:**

Older people exhibited mean age of 63.98 years, weight of 78.76 kg and height of 1.64 m. The mean CPITN score was 2.84. 31.7% of the study population were smokers and 53.8% females. No statistically significant difference was observed in seniors between periodontal treatment needs and systemic diseases. Females exhibited statistically significant more often osteoporosis, thyroid disorders (*p*<0.001) and hypercholesterolemia (*p*=0.014) than males. High CPTIN scores were not associated with higher levels of BMI.

**Conclusions:**

Within the limitation of this study, older adults’ periodontal treatment needs are not associated significantly with a great number of systemic diseases and body mass index.

** Key words:**Seniors, periodontitis, systemic diseases, body mass index (BMI), smoking, Greece.

## Introduction

Interest in oral health condition of older people has increased and research was conducted in both developing and developed countries in the last three decades. After the industrial revolution, death rate has decreased, and global human population has increased. Low population growth rate leads to population aging (1). Periodontal disease is a multifactorial inflammatory disease, which is associated with other chronic diseases. Nowadays, there is abundant evidence that connects the presence of periodontitis with certain systemic diseases, such as diabetes mellitus, cardiovascular disease, osteoporosis, respiratory disease, dementia, rheumatoid arthritis and cancer (2). Most of the evidence are based on epidemiological studies.

Diabetic individuals with uncontrolled glycemic levels are more susceptible to onset infectious diseases, such as periodontal disease. Also, patients with severe periodontal disease are more prone to have high hbA1c levels (3). Severe periodontitis is more prevalent in diabetics compared to non-diabetics, and the association between diabetics and non-diabetics has been described as 59.6%:39% (4). Both cardiovascular and periodontal diseases share common risk factors, and individuals with periodontal disease are at high risk for cardiovascular diseases. A meta-analysis concluded that patients with periodontal disease exhibit a 19% increased risk of cardiovascular disease (CVD) development (5). The relationship between osteoporosis and periodontal disease is still under examination. However, a great number of studies suggested that bone loss is related to skeletal osteoporosis. Martínez-Maestre and colleagues concluded in a systematic review that systemic osteoporosis was associated with radiological findings of osteoporosis in mandible and tooth loss (6). Other possible risk factors of periodontal disease are stress, depression and psychological factors (7).

Studies have also indicated that oral health conditions are related to quality of life. Impaired oral health results in malnutrition and weight loss (8). Inadequate nutrient intake and weight loss are factors related to frailty, vulnerability and mortality (9). Older people with oral disorders exhibit problems with pain, eating habits, chewing and social life. These conditions have significant influence on life satisfaction and psychological well-being, as described by Locker and colleagues (10).

The objectives of the present study were to evaluate:

1) The association between systemic diseases and periodontal treatment needs in an older population in Greece

2) The association between periodontal and body weight status.

3) Gender-related differences in presence of systematic diseases and in clinical measurements.

## Material and Methods

Participants were recruited from patients visiting the outpatient clinic of the School of Dentistry at Aristotle University of Thessaloniki, Greece during three months. A total of 262 individuals aged 55 years and more from a sample of 600 patients participated in this study. Clinical measurements, health histories and demographic data were obtained during the examination at the outpatient clinic. Exclusion criteria included: patients younger than 55 years; pregnant; crippled; and edentulous in one or both jaws.

All patients were carefully informed about the objectives of the study and gave written informed consent to sign. The study was reviewed and approved by the Aristotle University of Thessaloniki, Greece ethics committee in accordance with the Helsinki declaration on human studies.

-Health history and demographic data

All participants were asked to complete a health history questionnaire and then it was discussed with the dentists in order to record accurately the medical history. Also, all the medications taken by the patient were recorded. The presence of the following medical conditions were included in the study: CVD; diabetes mellitus; hypertension; hypercholesterolemia; osteoporosis; respiratory diseases; thyroid disease; hepatitis; psychological disorders; rheumatoid arthritis; and kidney disease. The taking medications were recorded in a case of these conditions.

Smoking habits were categorized as: non-smokers (patients who had never smoked or stopped smoking for more than five years); and smokers (patients who smoked daily or past smokers who had stopped smoking less than five years previously).

-Oral and body measurements

Oral examinations were assessed by a single investigator using a World Health Organization community periodontal treatment needs (CPITN) probe. The examiner was calibrated before the examinations. All subjects were evaluated clinically, and periodontal status was recorded for each sextant, when two or more teeth, not designated for extraction, were present; if only one tooth remained in the sextant, it was included in the adjoining sextant. The parameters were assessed at six sites around each tooth (mesio-buccal, mid-buccal, distobuccal, mesio-lingual, mid-lingual and disto-lingual locations), excluding third molars. Full mouth measurements were recorded. The total CPITN score was determined from the most severe measure in a sextant.

The weight of each individual was recorded in kilograms using a scale. As far as height is concerned, it was measured in centimeters using a vertical metric ruler. Both weight and height measurements were calibrated before the beginning of the study. Body Mass Index (BMI) was calculated as weight (in kilograms) divided by the square of height (in meters).

-Statistical analysis

Descriptive statistics such as frequencies, means, standard deviations, ranges and minimum-maximum scores were conducted using the IBM Statistics package version 21.0. In order to compare the means of CPITN scores between non-diseased and participants with systemic disease a series of Mann Whitney U tests were conducted. Clinical measurements were analyzed using logistic regression analysis to adjust gender, age and smoking habits in patients suffering from a systemic disease or not. Chi-square tests were also conducted to evaluate the statistical significance of CPITN scores between males-females and smokers-non-smokers as well. Statistical significance was set to 0.05. The correlation between body mass index (BMI) and CPITN scores was calculated using Spearman’s rank correlation coefficient.

## Results

-Descriptive statistics

From a total of 262 participants that constituted the sample of the current study, 121 (46.2%) were males, while 141 (53.8%) were females. Of the 262 subjects, 83 (31.7%) were smokers, whereas 179 (68.3%) were non-smokers ( Table 1). Mean subject characteristics were: age 63.98 years (std. deviation 7.29); weight 78.76 kg (std. deviation 17.47); height 1.64 m (std. deviation 0.09); and CPITN 2.84 (std. deviation 0.95) ( Table 2).

**Table 1:**
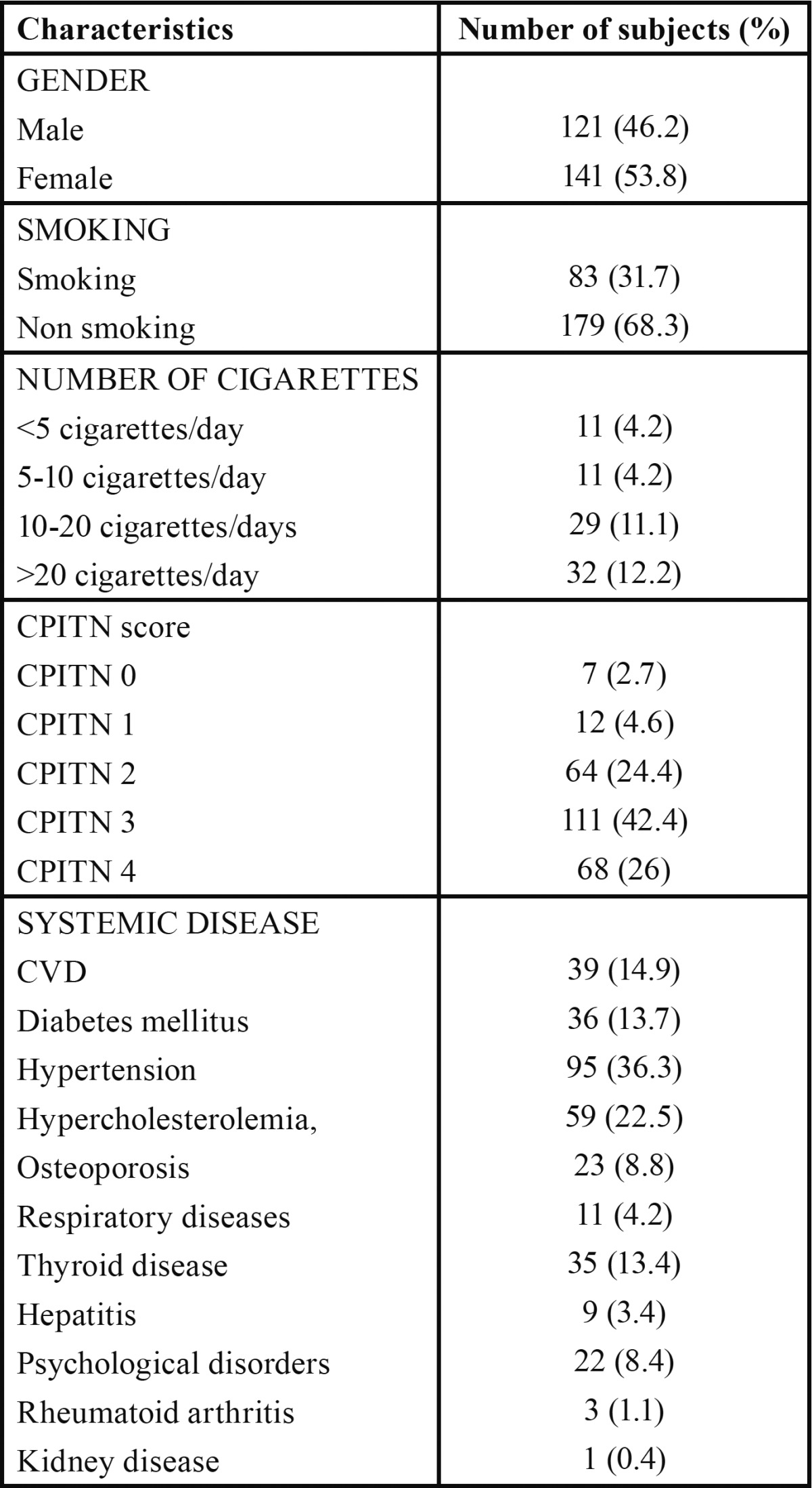
Basic characteristics of the study population.

**Table 2:**
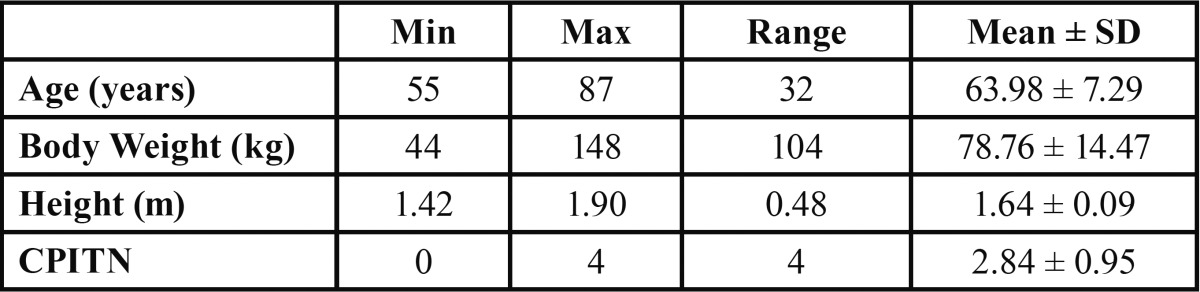
Numerical data of the study population.

The participants were divided into 11 groups according to the examined systemic diseases: CVD 39 (14.9%); diabetes mellitus 36 (13.7%); hypertension 95 (36.3%); hypercholesterolemia 59 (22.5%); osteoporosis 23 (8.8); respiratory diseases 11 (4.2%); thyroid disease 35 (13.4); hepatitis 9 (3.4%); psychological disorders 22 (8.4%); rheumatoid arthritis 3 (1.1%); and kidney disease 1 (0.4%) ( Table 1).

According to the periodontal measurements, using the CPITN probe, the majority of the population (179 individuals, 68.4%) exhibited increased treatment needs (CPITN 3-4) ( Table 1).

-Periodontal treatment needs and systemic diseases

Mann Whitney U tests were conducted to evaluate the impact of 11 different systemic diseases on the periodontal status. Periodontal status and treatment needs as assessed by using CPITN, were not associated with any systemic disease. There were no statistically significant differences in the periodontal conditions between diseased and non-diseased individuals. The mean ± SD scores of CPITN are presented in  Table 3. The sample population are divided into groups according to their systemic condition: diseased or non-diseased from CVD, diabetes mellitus, hypertension, hypercholesterolemia, osteoporosis, respiratory diseases, thyroid disease and psychological disorders. As presented in  Table 3, all the examined systemic diseases exhibited comparable odds ratios. This indicates that individuals suffering from one of these diseases displayed the same treatment needs with non-diseased patients. In the same way, non-significant association was observed between non-diseased individuals and subjects suffering from more than one of the included systemic diseases (*p*=0.18).

**Table 3:**
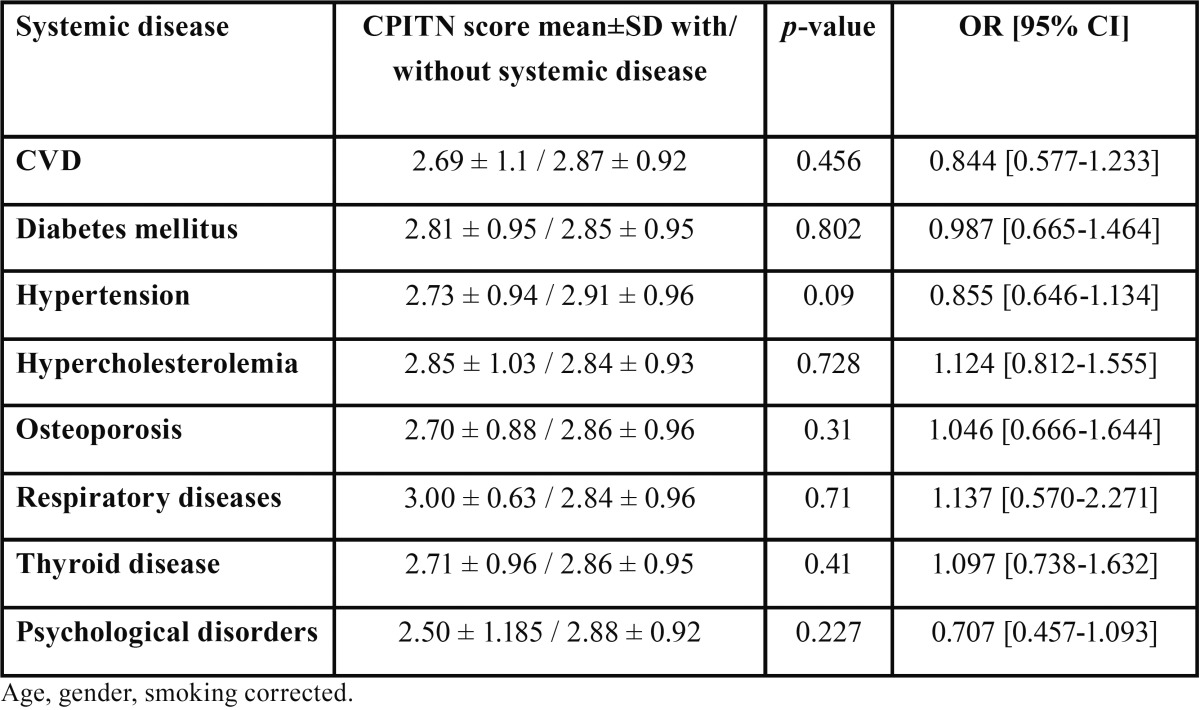
Clinical measurements and systemic diseases.

-Periodontal and body mass index (BMI)

The relationship between body mass index (BMI) and CPITN score was investigated using the Spearman’s rank correlation coefficient. There was no correlation between the two variables, r=.042, n=262, *p*=0.499, with high CPTIN scores not associated with higher levels of BMI.

-Gender-related differences 

Males and females were compared to the occurrence of systemic diseases. Females exhibited statistically significant more often osteoporosis (15.6% females and 0.8% males, *p*<0.001), thyroid disorders (24.1% females and 0.8% males, *p*<0.001) and hyper-cholesterolemia (28.4% females and 15.7% males, *p*=0.014) than males. The remaining systemic diseases did not show differences between men and women ( Table 4).

**Table 4:**
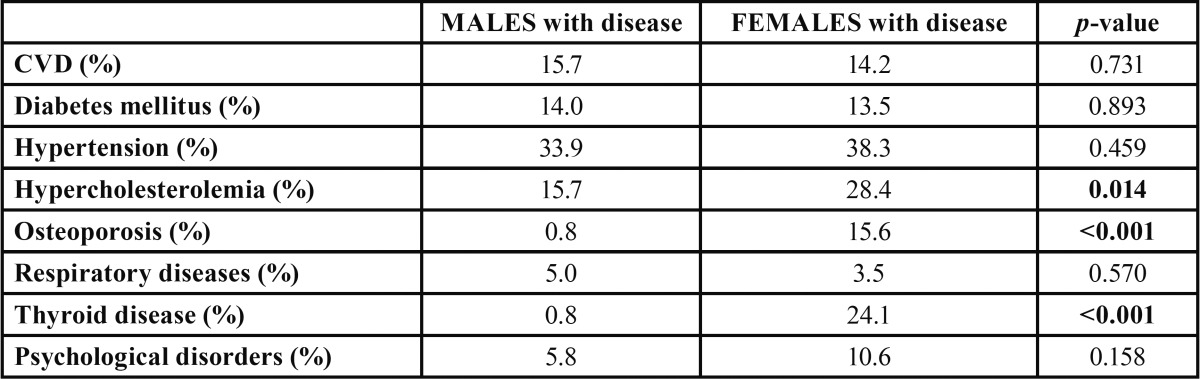
Gender differences in the presence of systemic disease.

The male population of the study differed significantly in relation to women regarding age (*p*=0.002), height (*p*<0.001) and weight (*p*<0.001). Smoking habits were comparable in both sexes (*p*=0.477) ( Table 5).

**Table 5:**
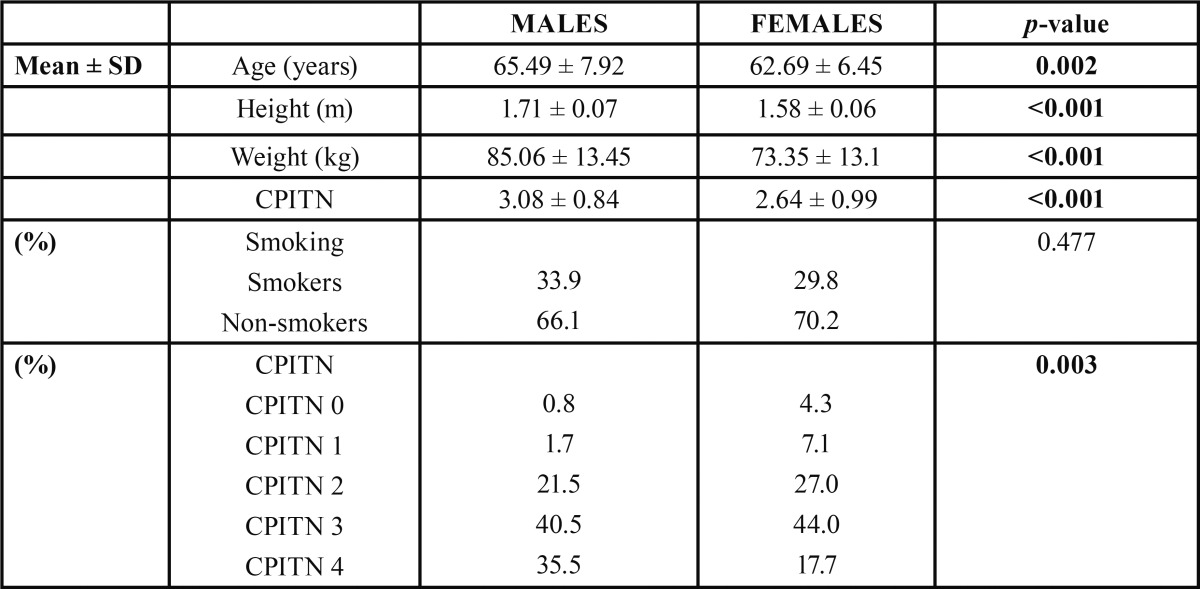
Gender differences on clinical measurements, age, smoking habits.

Men and women exhibited also significant differences regarding periodontal conditions. Men displayed higher mean CPITN scores (mean±SD= 3.08±0.84) than women (mean±SD= 2.64±0.99). 35.5% of men needed complex periodontal treatment (CPITN 4), whereas only 17.7% of women had the same needs. Hence, CPITN scores were significantly higher in men (*p*=0.003) ( Table 5).

-Smoking habits and periodontal status

Smoking influenced the severity of periodontal treatment needs of the older population. Smokers (mean±SD= 3.05±0.97) measured to have higher periodontal treatment needs than non-smokers (mean±SD=2.75±0.93). 38.6% of smokers had pockets 6 mm deep or deeper (CPITN 4), compared to only 20.1% of non-smokers. The observed differences were statistically significant (*p*=0.038).

## Discussion

Periodontal disease is a chronic disease caused by bacteria found in plaque. The immune response determines the disease progress. Doubtless, systemic diseases affect host defense and periodontal disease extension and severity. However, it is generally believed that it is difficult to determine the relationship between systemic factors and periodontitis (11).

The main purpose of this study was to assess the relationship between systemic diseases and periodontal status in a randomized sample of older adults examined in a university dental clinic in Greece during a period of financial crisis. During economic crisis the socio-economic inequalities have increased resulting injustices in health service accessibility (12). Income and educational level affect the access to healthcare services and the onset of chronic disease as well (13).

Despite the findings of several studies that systemic diseases are associated with periodontal diseases, in our study the examined systemic conditions did not affect the periodontal treatment needs significantly. Individuals, who were suffering from systemic conditions such as CVD, diabetes mellitus, hypertension, hypercholesterolemia, osteoporosis, thyroid disease and psychological disorders demonstrated similar periodontal treatment needs with non-diseased patients. Periodontitis, as a chronic inflammatory disease, may share common risk factors with other conditions. Age, gender, smoking, socio-economic status, attitude and other factors may affect the diseases (14).

Grossi and colleagues claimed that diabetics are more prone to attachment loss than non-diabetics with odds ratio 2.32 (15). However, older subjects may display analogous odds in periodontal disease progression, regardless of the presence of diabetes mellitus (16). Periodontal disease is associated with CVD due to inflammatory process and host defense. Periodontitis is an important risk factor for CVD such as stroke (17). Moreover, cross-sectional studies concluded that hypertension is associated with periodontal disease (18) and higher CPITN scores (19). However, no association was detected between CVD and hypertension with periodontitis in our study.

Psychological disorders may influence periodontal tissues. Several studies demonstrated that depression and stress are associated with onset and progression of periodontitis (20), as well as reduced oral hygiene (21). In older subjects, mean aged 67.2 years, depression was not correlated with the periodontal condition, but only with tooth loss (22). Osteoporosis is a common chronic disease, which affects mostly older women. In our study, 23 patients (8.8%) reported that they were diagnosed with osteoporosis, but they had similar treatment needs with participants having adequate density and quality of bone. Statistically significant gender-related differences in terms of osteoporosis were found in the present study: 22 women (95.7%, *p*<0.001). In another study of 1084 subjects aged 60-75, osteoporosis was associated with periodontitis with odds ratio 1.8 (95%CI: 1.2, 2.5, *p*<0.001) (23).

Generally, smoking is a well-known risk factor of periodontal disease. In our study, 31.7% were smokers and they were more prone to have deep periodontal pockets and complex periodontal treatment needs. This difference was statistically significant. The association between smoking habits and periodontal disease was also confirmed by other studies (24).

In a similar study published by Ozcaka and colleagues (25), CPITN scores and tooth loss were not correlated with the presence of systemic diseases. However, only patients undertaking antihypertensive drugs were more prone to tooth loss than patients that did not receive this medication. The same results occurred in our study as regards to the periodontal condition and the presence of systemic disease: no association found between systemic diseases and treatment needs. In addition, gender discrepancies were observed in 201 subjects from Western Turkey (25), with more treatment needs for men compared to women. Similar differences were recorded also in our study respecting gender and periodontal treatment needs: male population exhibited statistically significant higher treatment needs than females.

Another hypothesis posited that patients with higher treatment needs would exhibit higher nutritional problems compared to patients with low periodontal treatment needs. Nutritional deficiencies may lead to a reduced body mass index. However, in the present study, BMI and periodontal status were not correlated (*p*=0.499). In another study, Perera and Ekanayake (26) concluded that in older adults, missing teeth were correlated with underweight significantly. In another cross-sectional study with Brazilian seniors, patients without dental prosthesis and complete absence of teeth were associated with abnormal weight (underweight odds ratio= 3.94, 95% CI 1.14-13.64 or overweight/obesity OR = 2.88, 95% CI 1.12-7.40) (27).

An oral-systemic disease interaction model has been proposed to present the relationship between periodontal status and systemic condition. Periodontal and systemic diseases may have a two-way relationship. Not only the role of periodontal disease is important as an outcome of systemic diseases, but also as a risk factor for further systemic disorders (28). Elimination of periodontal inflammation could diminish the risk of systemic diseases. However, further studies are needed to show such an influence (29). Prevention plays a pivotal role in reducing the risk of periodontitis. Oral health and quality of life (dental pain, failure of communication and nutrition) are associated with oral hygiene significantly (30).

## Conclusions

Despite the limitation of the study population, the survey concluded that there was no significant difference between systemic diseased and non-diseased individuals regarding their periodontal treatment needs. Periodontal status of the examined older people was not influenced by the body mass index in the present study. However, sex-related and smoking-related differences were detected. Male and smoking population were examined with higher treatment needs compared to females and non-smokers respectively. Large national-level surveys are needed to be conducted in order to present the whole picture of seniors’ periodontal status.
